# The effect of individual sulfasalazine administration and in combination with esomeprazole on buccal mucosa of albino rats: histological, immunohistochemical, and molecular study

**DOI:** 10.1007/s10266-026-01350-2

**Published:** 2026-03-21

**Authors:** Mona Bahaa Morsy, Marwa M. Abd Elhameed, Reham Ahmed Mohamed Abdrabo, Safaa Ismail Hussein

**Affiliations:** 1 Department of Oral Biology, Faculty of Dentistry, Ain Shams University, El-Qobba Bridge, Al Waili, Cairo, 11566 Egypt; 2 Oral Biology Department, Field of Dentistry, Dentistry Program, Galala University, Attaka, 43511 Suez Governorate Egypt; 3 Oral Biology Department, Faculty of Oral and Dental Medicine, Egyptian Russian University, Cairo, Egypt

**Keywords:** Esomeprazole, Sulfasalazine, Ferroptosis, Buccal mucosa, Immunohistochemistry

## Abstract

The study aims to investigate the effect of sulfasalazine (SFZN) individually and in combination with esomeprazole (ESOm) on the buccal mucosa of albino rats. SFZN is a widely used drug for the management of various autoimmune diseases which has been reported to cause renal injury in humans and a dose of 600 mg/kg/day has been described to cause renal injury in rats. ESOm is a commonly used proton pump inhibitor. Few studies have investigated their effects on oral and paraoral tissues. Three groups were designed out of 27 male albino rats. The control group was given distilled water, the SFZN group was given SFZN (600 mg/kg/day), and the ESOm group was given SFZN (600 mg/kg/day) and ESOm (30 mg/kg body weight); the drugs were dissolved in distilled water. The experiment was conducted for 14 days. Buccal mucosae were evaluated for keratin thickness, area % of iron deposition, area% of immunoreactivity to nuclear factor erythroid 2-related factor 2 (Nrf2) and glutathione (GSH) tissue level using one-way ANOVA and post hoc tests. The level of statistical significance was set at *p* < 0.05. SFZN individually has significantly increased keratin thickness, disrupted Nrf2 machinery and glutathione tissue level, while SFZN in combination with ESOm showed significant increase in area% of iron deposition. DNA degradation using comet assay was evaluated using Kruskal–Wallis and Dunn’s test which revealed no significant difference of tail length and tail moment in SFZN group and ESOm group but these parameters were significantly different in these two groups in relation to the control group (*p* < 0.05).

**Graphical abstract:**

Effect of sulfasalazine individually and in combination with esomeprazole on buccal mucosa of albino rats evaluated via morphometric analysis for keratin thickness, tissue iron deposition and immunoreactivity to Nrf2 and via molecular analysis measuring glutathione tissue level and DNA degradation (This figure has been created using BioRender.com)

**Figure:**
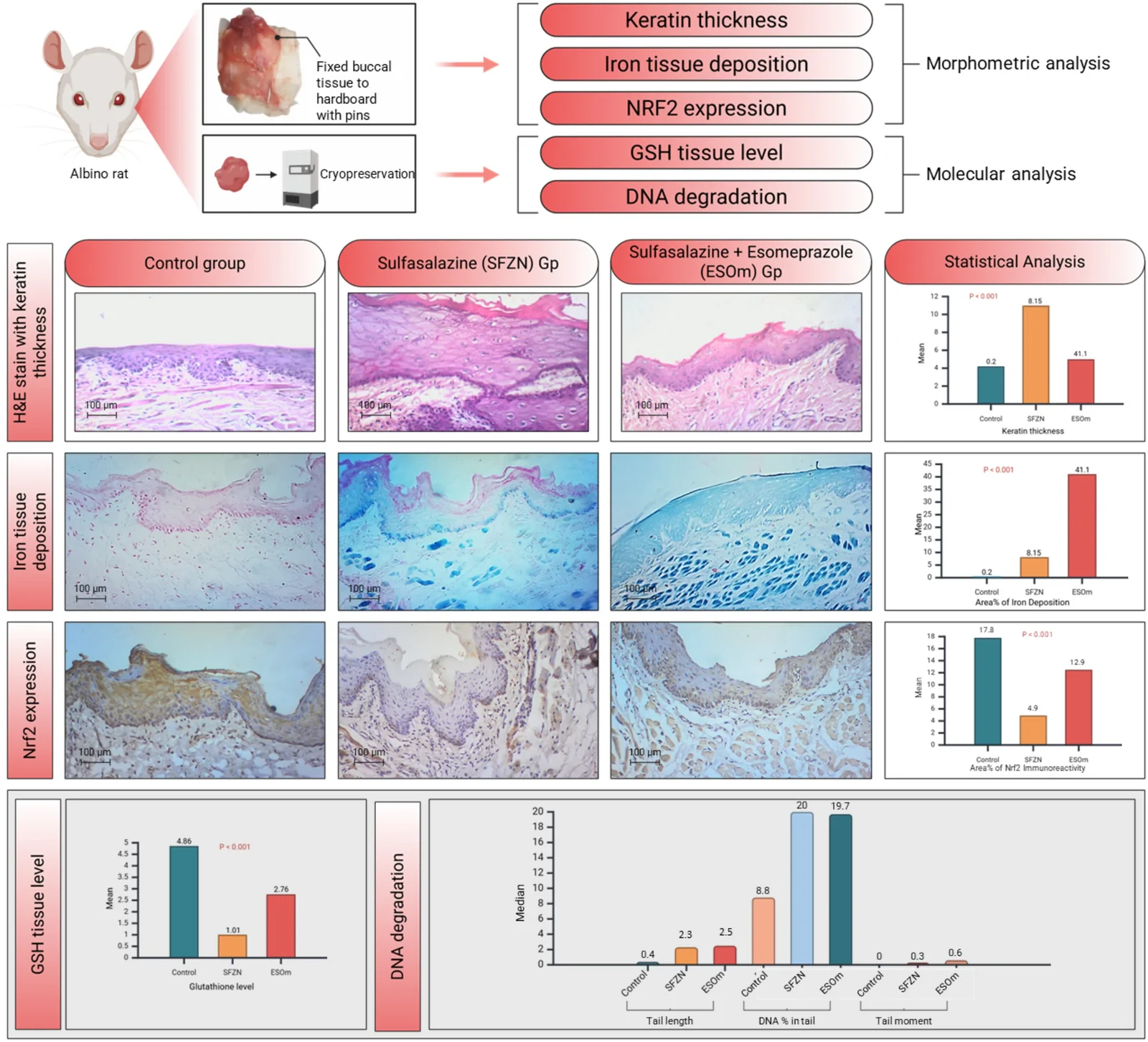

## Introduction

Sulfasalazine (SFZN) is widely used in the management of ulcerative colitis, inflammatory bowel diseases, and rheumatoid arthritis in humans [1].

It has been linked to ferroptosis, a regulated cell death mainly caused by iron-mediated lipid peroxidation, leading to membrane damage and cell lysis. It is an Erastin derivative that has been documented to inhibit glutathione peroxidase 4 (GPX4). Erastin inhibits system xc^**−**^, an ATP-dependent cystine/glutamate antiporter that can import a single cystine molecule into the cells and export glutamic acid out of the cells. It is a heterodimer composed of SLC7A11 (cystine transporter solute carrier family 7 member 11) and SLC3A2 (Solute Carrier Family 3 Member 2), linked by disulfide. Inhibition of this system leads to depletion of cystine with subsequent depletion of GSH (Glutathione), a cofactor of GPX4, that is required for the ferroptosis-resistance activity of GPX4 [2, 3].

Epigenetic, transcription, and post-translational mechanisms control a variety of molecular signaling pathways that control ferroptosis [4]*.*

Ferroptosis can be considered a double-edged weapon as it suppresses the growth of many types of tumors. Furthermore, it was believed to improve the efficacy of chemotherapy, radiotherapy, or immunotherapy [5].

Esomeprazole (ESOm) is a proton pump inhibitor, used to treat duodenal and gastric ulcers, erosive esophagitis, gastroesophageal reflux disease, and Zollinger–Ellison syndrome. It was reported to reverse hepatocellular damage and confer significant anti-fibrotic actions [6]. The therapeutic efficacy of ESOm plus SFZN was reported by Balza et al. (2017) on certain types of sarcoma [7]. ESOm and SFZN, in combination, were reported to diminish endothelial dysfunction and to be a potential combination treatment for preeclampsia [8].

To our knowledge, there is a lack of studies investigating the effect of SFZN individually and in combination with ESOm on the buccal mucosa of albino rats. The buccal mucosa exhibits rapid healing, is highly vascularized, and resides in a bacteria-rich environment [9]. Our study marks the first exploration of the impact of SFZN individually and in combination with ESOm on the buccal mucosa of albino rats, which has possible clinical relevance and possible management of patients in need of such treatments.

## Materials and methods

### Drugs

Sulfasalazine (500 mg tablets) was purchased from ACDIMA, Co., Egypt, and esomeprazole (10 mg sachets) was purchased from AUG, Co., Egypt.

### Experimental animals

The ethics committee at Ain Shams University Faculty of Dentistry (FDASU-REC) approved the experimental work with authorization number [FDASU-Rec ID032402] and offered ethical guidelines for the investigation. The experiment was held in the Faculty of Medicine, Ain Shams Research Institute (MASRI).

Power analysis was performed to determine the sample size using the Epicalc program version 1.02, assuming a power of 80% and alpha = 0.05 based on a previous study conducted by Niknahad et al. (2017) [1].

Twenty-seven adult male albino rats weighing 200–250 g were kept in cages made of metal mesh, with a maximum of five rats per cage, under controlled circumstances, following the Ethical Committee’s predetermined requirements.

### Experimental design

Nine rats were assigned to each one of the following groups: Control group, sulfasalazine group (SFZN Gp), and sulfasalazine + esomeprazole group (EZOm Gp). Distilled water was given to the Control group. For the SFZN Gp, sulfasalazine (SFZN) suspension (600 mg/kg/day) was prepared by grinding the tablets and dissolving them in distilled water and was given to the rats once daily using an oral gavage for 14 consecutive days [1, 10]. SFZN has been reported to cause renal injury in humans and a dose of 600 mg/kg/day has been described to cause renal injury in rats [1], the oral effect of this dose on buccal mucosa has not to our knowledge been described before.

For the EZOm Gp, SFZN suspension was given to them in addition to esomeprazole (ESOm) suspension prepared freshly by dissolving ESOm sachets in distilled water according to the manufacturer’s instructions, 30 mg/kg body weight [6]. An oral gavage was used for the ESOm suspension, and the dose was given to the rats once early in the morning before giving them any diet for 14 consecutive days*.*

### Preparation of histopathological sample

Our experiment ended with the anesthetic overdose given to the animals. The buccal mucosae of all groups were dissected. The left side was fixed in 10% buffered formalin for 48 h. Following fixation, specimens were prepared at the pathology department, Faculty of Medicine, Ain Shams University, to be stained with hematoxylin and eosin (H&E), Mallory Prussian blue special stain [11], and an immunohistochemical stain to be viewed under a light microscope.

### Histochemistry

To determine the level of intracellular or mitochondrial ferrous iron and Fenton reaction, 4–6µ sections were processed and stained with (Mallory Prussian blue) [11].

### Immunohistochemistry staining protocol

Sections were incubated with a polyclonal antibody against Nrf2 (Novus, NBP1-32822) according to the manufacturer’s instructions and as previously described [12].

### Preparation of samples for DNA degradation analysis and glutathione (GSH) tissue level

The right side of the buccal mucosa in all groups was excised and divided into two halves that were cryopreserved in ice-cold medium and transferred as soon as possible to the National Institute for Animal Research to measure GSH buccal tissue level using colorimetric methods (Bio-Diagnostics Ltd, Egypt) [13], which scientific research uses to measure oxidative stress levels in biological samples.

### DNA degradation analysis

Single cell gel electrophoresis, the COMET assay, was performed [14].

### Digital analysis and morphometric analysis

The morphometric study for the H&E results, Mallory Prussian blue special stain and immunohistochemical results was performed using the computerized image analysis system (Leica QWin software, Leica Microsystems, Germany) in the Department of Oral and Dental Pathology, Faculty of Dental Medicine for Girls, Al-Azhar University, by expert pathologists, to measure the keratin thickness in each group and the percent of area showing reaction to Prussian blue stain [15, 16], and to Nrf2 immunohistochemical stain[17, 18].

### Statistical analysis

The collected data were revised, coded, tabulated, and introduced to a PC using the Statistical Package for the Social Sciences (SPSS 15.0 for Windows; SPSS Inc., Chicago, IL, 2001). One-way ANOVA and Fisher’s least significant difference (LSD) post hoc tests were used for statistical evaluation of keratin thickness, area % of iron deposition, area% of immunoreactivity to Nrf2 and GSH tissue level. The LSD test was chosen because only three groups were compared. Kruskal–Wallis and Dunn’s test were used for evaluation of comet assay results. P-values less than 0.05 were considered statistically significant differences (SSD).

## Results

### H&E results (defective epithelialization and increased keratin thickness in SFZN Gp)

**Fig. 1:**
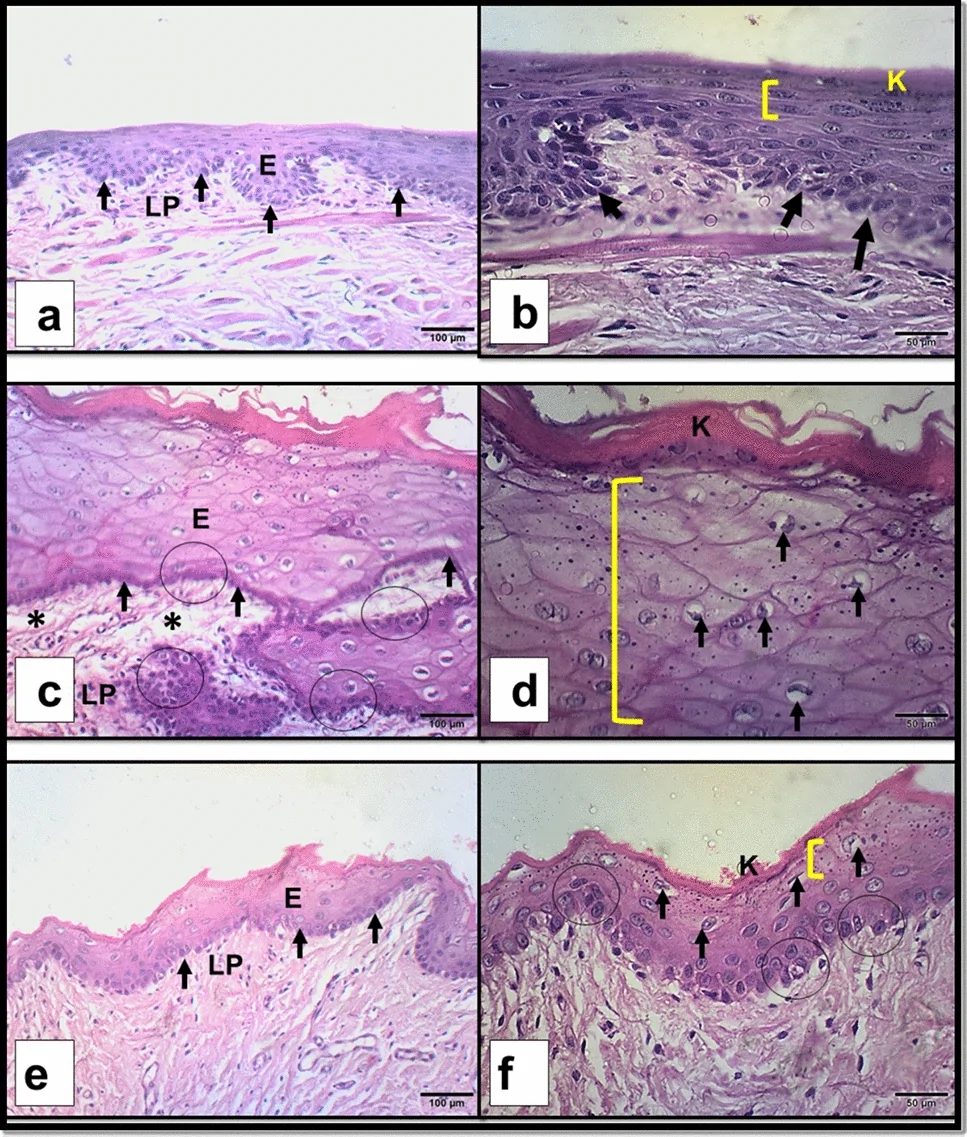
Photomicrographs of the H&E-stained buccal mucosa: **a** represents the control Gp and shows normal keratinized stratified squamous epithelium (E) with well-defined BM (arrows) and normal LP. **b** is a higher magnification showing columnar basal cells (arrows), granular cell layer confined to the superficial layers of epithelium (bracket), and well-defined keratin layer (K). **c** represents the SFZN Gp and shows rounding of basal cells (circles), apparently increased epithelial thickness (E), with irregular BM (arrows) with loss of rete pegs. Note the separation of collagen fibers in LP (Asterisk). **d** is a higher magnification for SFZN Gp showing stratum granulosum occupying nearly half of the epithelial thickness(bracket) and increased keratin thickness (K). Note the vacuolated cells with pyknotic and pleomorphic nuclei (arrows). **e** represents the ESOm Gp and shows epithelial thickness comparable to control and regular BM. LP showing dense fibrous CT. **f** is a higher magnification showing rounding of the basal cells to some extent (circles), stratum granulosum (bracket) confined to the superficial layer of the epithelium, vacuolation and pleomorphic nuclei (arrows) (H&E, a, c, e Org.mag × 200, b, d, f Org.mag × 400)

Histological examination of the control group showed normal histological features with apparent normal epithelial thickness. The epithelium and lamina propria (L.P) were separated by a well-defined Basement membrane (B.M). The epithelial rete pegs appeared short and broad. The keratin layer appeared to be a well-defined acidophilic layer covering the epithelium. The epithelium was underlined by normal fibrous connective tissue (C.T). The L.P consisted of dense vascular C.T with fibroblasts and collagen fiber bundles (Fig. 1a). Higher magnification revealed the different layers of the epithelium showing normal columnar basal cells, polygonal prickle cell layer, and granular cell layer confined to the superficial layers of the epithelium (Fig. 1b).

The SFZN Gp showed an apparent increase in thickness compared to the control Gp, together with vacuolated cells, irregularities of the BM were visible, and marked separation in the collagen fibers of the LP (Fig. 1c). Higher magnification showed rounding of the basal cells, pyknotic and pleomorphic nuclei in the prickle cell layer, premature keratinization represented by a granular cell layer occupying nearly half of the epithelium thickness, and irregular increased keratin thickness (Fig. 1d).

The ESOm Gp showed some vacuolization, but the epithelial thickness appeared almost normal, the BM and the rete pegs appeared comparable to the control Gp, and the LP showed regular condensed CT (Fig. 1e). The deteriorated histological findings in the SFZN Gp appeared to be restored in the ESOm Gp. Higher magnification revealed the characteristic rounded appearance of the basal cell layer, some degree of vacuolation, and nuclear pleomorphism related to the granular cell layer, which appeared confined to the superficial layers of the epithelium, comparable to that shown in the control group (Fig. 1f).

The one-way ANOVA test was performed to evaluate the keratin thickness in all groups, followed by LSD post hoc test (the LSD test was chosen because only three groups were compared), which revealed that the mean of keratin thickness in the SFZN group was the highest, followed by the ESOm and Control groups. This result was statistically highly significant. There was statistically significant difference (SSD) between {SFZN vs. (Control & ESOm)}. While, there was no SSD between {Control vs. ESOm}. The mean difference of Keratin thickness for SFZN Gp was higher than those for Control and ESOm by 6.8 and 6.1, respectively (Tables 1, 2, Fig. 4a).

**Table 1:** Keratin thickness in H&E sections, area % of iron deposition, area % of reaction to NRF2, and GSH buccal tissue level

	Control Gp	SFZN Gp	ESOm Gp	F	P value
Keratin thickness in H&E sections	4.2 ± 0.8 ^a, c^	11.0 ± 4.0 ^b^	5.0 ± 0.8 ^c, a^	24	< 0.001 ^**^ (HS)
Area % of iron deposition	0.20 ± 0.17 ^a^	8.15 ± 5.02 ^b^	41.10 ± 7.16 ^c^	185	< 0.001 ^**^ (HS)
Area % of reaction to NRF2	17.8 ± 4.0 ^a^	4.9 ± 2.7 ^b^	12.5 ± 1.7 ^c^	48.3	< 0.001 ^**^ (HS)
GSH buccal tissue level	4.86 ± 0.23 ^a^	1.01 ± 0.07 ^b^	2.76 ± 0.86 ^c^	141	< 0.001 ^**^ (HS)

One-way ANOVA test, different letters = S, same letters = NS, P > 0.05 = NS, P < 0.01 = HS**, P < 0.05 = S *. Values are displayed as means with standard deviations ± SD (n = 9)

**Table 2:** Post hoc pairwise multiple comparison between groups regarding keratin thickness in H&E sections, area % of iron deposition, area % of reaction to NRF2, and GSH buccal tissue level

	Group (I)	Group (J)	Mean difference (I-J)	P value	Sig
*Keratin thickness*	*SFZN Gp*	*Control Gp*	6.8	< 0.001 ^**^	HS
		*ESOm Gp*	6.1	< 0.001 ^**^	HS
	*ESOm Gp*	*Control Gp*	0.7	0.506	NS
*Glutathione*	*Control Gp*	*SFZN Gp*	3.9	< 0.001 ^**^	HS
		*ESOm Gp*	2.1	< 0.001 ^**^	HS
	*ESOm Gp*	*SFZN Gp*	1.8	< 0.001 ^**^	HS
*Area % iron deposition*	*ESOm Gp*	*Control Gp*	40.9	< 0.001 ^**^	HS
		*SFZN Gp*	33.0	< 0.001 ^**^	HS
	*SFZN Gp*	*Control Gp*	7.9	0.002 ^**^	HS
Area %	*Control*	*SFZN Gp*	12.9	< 0.001 ^**^	HS
		*ESOm Gp*	5.3	< 0.001 ^**^	HS
	*ESOm Gp*	*SFZN Gp*	7.6	< 0.001 ^**^	HS

One-way ANOVA (Fisher’s LSD post hoc)

### Mallory Prussian blue special stain results (increased tissue iron deposition in SFZN Gp and ESOm Gp)

**Fig. 2:**
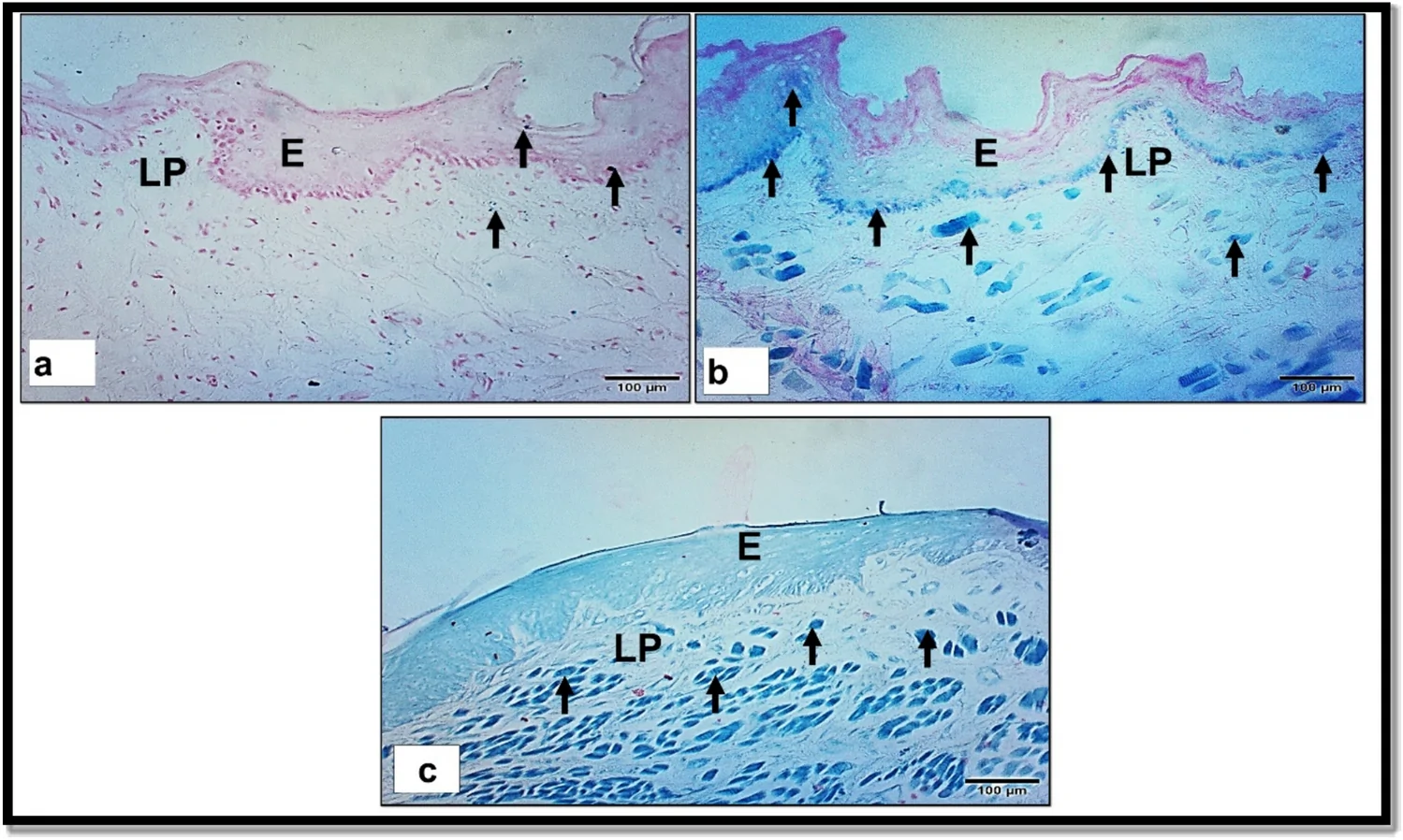
Photomicrographs of buccal mucosa stained with Mallory Prussian blue: **a** represents the control Gp and shows very mild iron deposition in the epithelium (E) and LP (arrows). **b** represents the SFZN Gp and shows moderate iron deposition in the epithelium (E) and LP (arrows). **c** represents the ESOm Gp and shows generalized very strong iron deposition in the epithelium (E) and LP (arrows) (Mallory Prussian blue, Org.mag × 200)

The control group showed mild iron deposition in the covering epithelium and the underlying lamina propria (Fig. 2a). The SFZN Gp showed moderate iron deposition in the covering epithelium and the underlying LP (Fig. 2b). The ESOm Gp showed strong iron deposition in the covering epithelium and the underlying L.P (Fig. 2c).

A one-way ANOVA test was performed, followed by LSD post hoc test, which revealed that the mean of ESOm Gp was the highest, followed by SFZN Gp and the control Gp. These results were statistically highly significant. There was SSD between {ESOm vs. Control & SFZN} & {SFZN vs. Control}. The mean of area% of iron deposition for ESOm Gp was higher than those for the control and SFZN groups by 40.9 and 33, respectively, and vice versa, while the mean of area% of iron deposition for SFZN Gp was higher than that for the control Gp by 7.9, respectively (Table 1, 2, Fig. 4b).

### Immunohistochemical results (downregulation of Nrf2 in SFZN Gp and ESOm Gp)

**Fig. 3:**
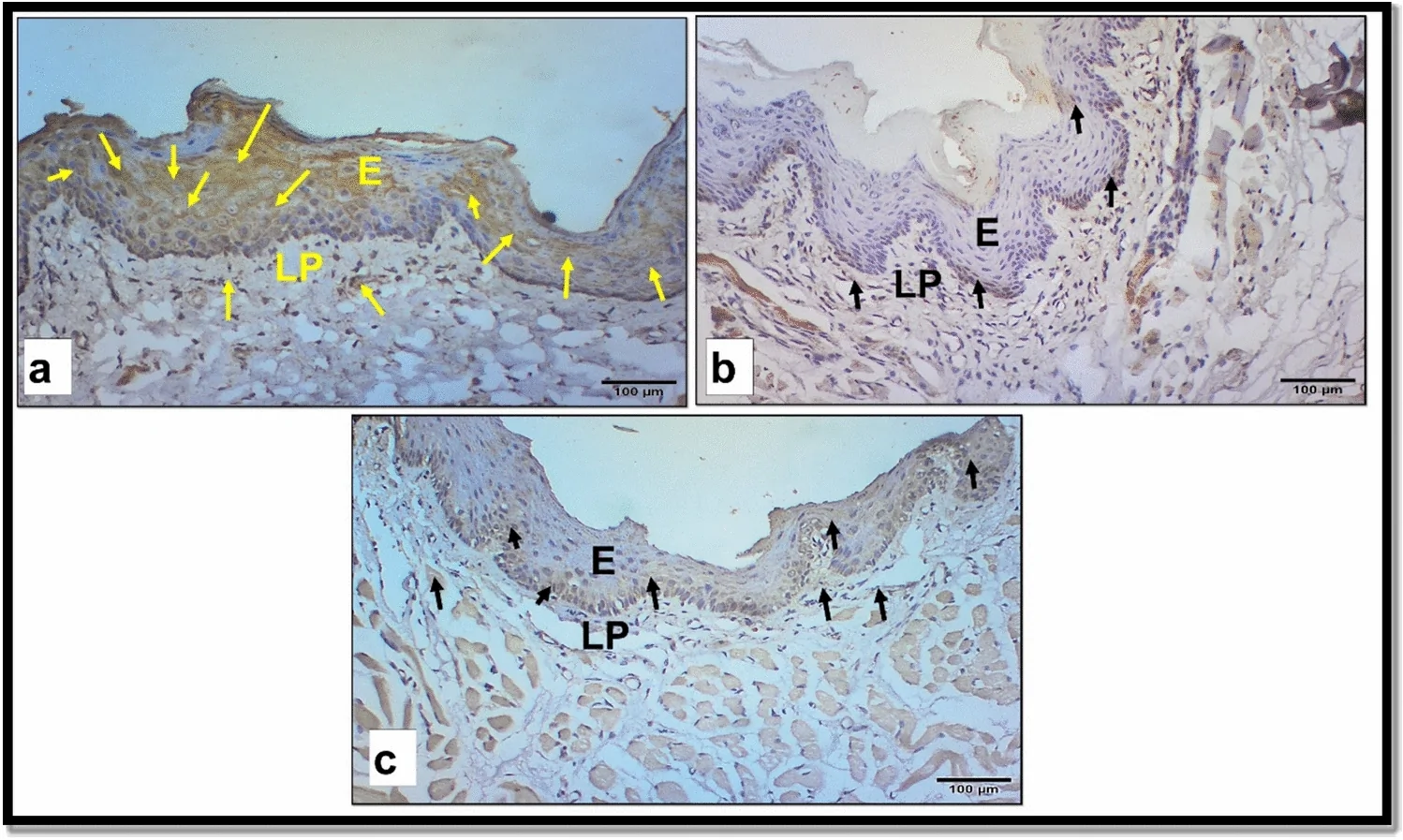
Photomicrographs of buccal mucosa: **a** represents the control Gp and shows strong immunoreactivity to Nrf2 in the epithelium (E) and LP (arrows). **b** represents the SFZN Gp and shows mild positive expression in the epithelium (E) and LP (arrows). **c** represents the ESOm Gp and shows moderate expression in the epithelium (E) and LP (arrows) (magnification × 200)

The control group showed strong positive reactivity toward NRF2 in the epithelial layer and L.P (Fig. 3a). On the other hand, the SFZN Gp showed mild immunoreactivity in the covering epithelium and underlying L.P (Fig. 3b), while the ESOm Gp showed moderate positive immunoreactivity to NRF2 in the covering epithelium and underlying L.P (Fig. 3c).

**Fig. 4:**
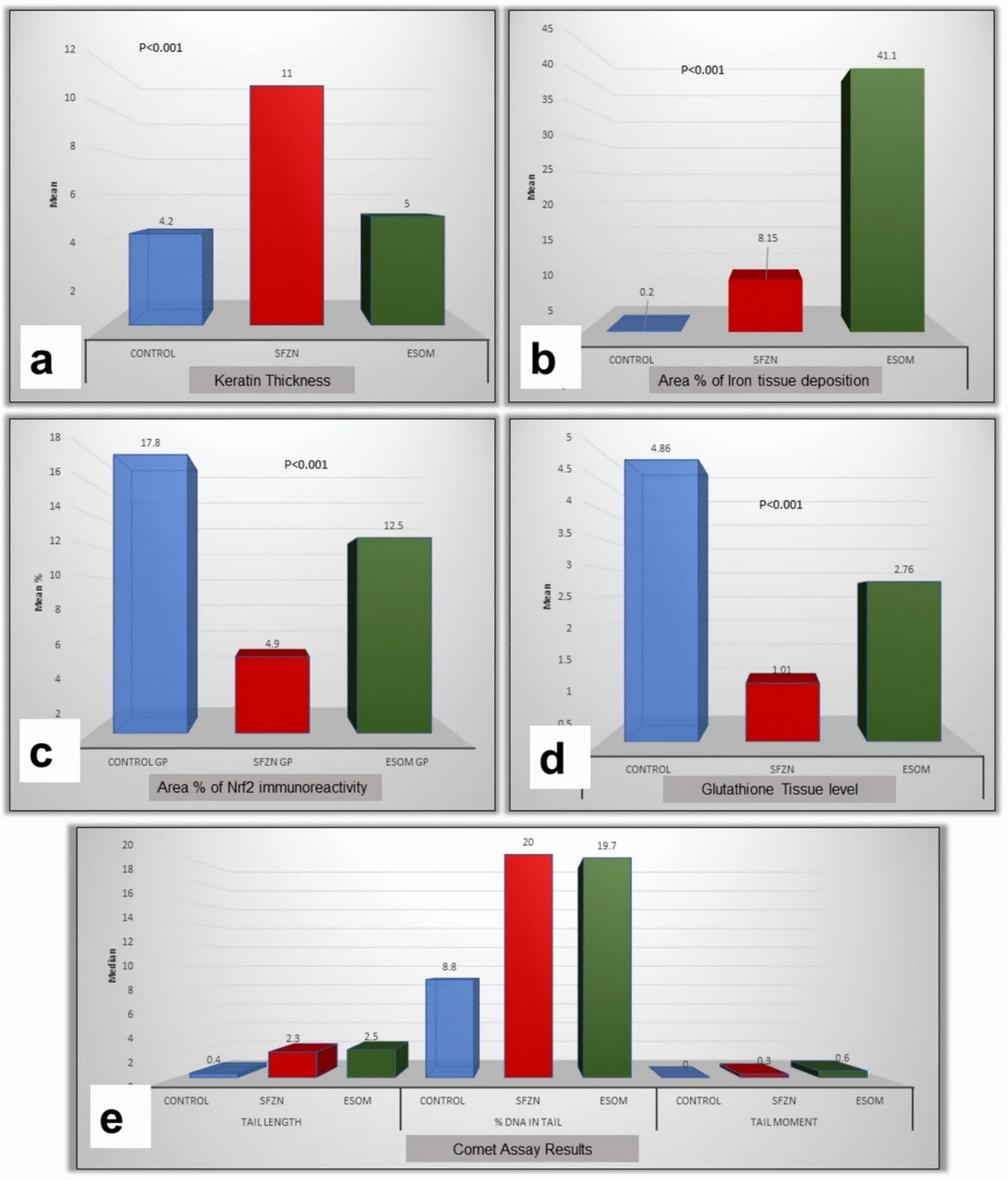
The bar charts represent comparison between the groups regarding **a** keratin thickness, **b** area % of iron deposition, **c** area % of immunoreactivity to NRF2, **d** glutathione tissue level, and **e** tail length, %DNA in tail, and tail moment of the comet assay

A one-way ANOVA test was performed, followed by LSD post hoc test, which revealed that the mean of the control Gp was the highest, followed by ESOm and SFZN groups. These results were statistically highly significant between the three groups. There was SSD between {control *vs.* SFZN Gp & ESOm Gp}. In addition, there was SSD between {ESOm Gp *vs.* SFZN Gp}. The mean of area% of control was higher than those for SFZN Gp and ESOm Gp by 12.9% and 5.3%, respectively, and vice versa. Moreover, the mean of area % for ESOm Gp was higher than that for SFZN Gp by 7.6% (Table 1, 2, Fig. 4c).

### Depletion of glutathione tissue level (GSH) in SFZN Gp and ESOm Gp

Results obtained from the three groups in mmol/g tissue were tabulated and transferred to Excel sheets for statistical analysis. A one-way ANOVA test, followed by LSD post hoc test, was performed and revealed that the mean for the control Gp was the highest, followed by ESOm and SFZN groups, respectively, and these results were statistically highly significant between the three groups. There was SSD in GSH levels between {Control vs. (SFZN & ESOm)} & {ESOm vs. SFZN}. The mean difference of GSH for control Gp was higher than those for SFZN and ESOm by 3.9 and 2.1 and vice versa, while the mean of ESOm Gp was higher than that for SFZN Gp by 1.8 (Table 1, 2, Fig. 4d).

### DNA degradation analysis by comet assay results (single-stranded DNA damage in SFZN Gp and ESOm Gp)

The variable data regarding tail length, tail moment, and DNA % in tail were obtained from the three groups, tabulated, and transferred to an Excel sheet for statistical analysis. The tail length, tail moment (calculated as a product of the tail DNA% and tail length), and tail DNA% are the three most commonly used measures in the comet assay to evaluate DNA migration [19]. Jiang et al. (2023) [20] reported that many scholars recommend relying on the tail moment in evaluating DNA migration. Alkaline comet assay conditions detect mostly single-stranded DNA breakage, while neutral comet assay conditions detect mostly double-stranded DNA breakage [21]. The electrophoresis conditions of the comet assay in our study were performed in alkaline PH conditions according to Singh et al. (1988) [14]. The comet analysis results of our study reflected significant DNA damage in both SFZN Gp and ESOm Gp, particularly single-stranded DNA damage.

For statistical analysis, the Kruskal–Wallis test followed by Dunn’s test was performed and revealed that the median of ESOm Gp was the highest, followed by SFZN Gp and control Gp, regarding tail length and tail moment. These results revealed no SSD between the ESOm Gp and SFZN Gp, while there was SSD between the two groups and the control group. On the other hand, there was no SSD between the three groups regarding DNA% in the tail (Table 3, 4, Fig. 4e).

**Table 3:** Comet assay parameters (*tail length*, *% DNA in tail*, *tail moment)*

	Control Gp	SFZN Gp	ESOm Gp	Kruskal–Wallis	P value
*Tail length*	0.4 ± 3.0 ^a^	2.3 ± 11.3 ^b, c^	2.5 ± 8.5 ^c, b^	9.4	0.009 ^**^(HS)
*Tail moment*	0.0 ± 1.5 ^a^	0.3 ± 7.3 ^b, c^	0.6 ± 5.3 ^c, b^	6.9	0.031 ^*^(S)
*% DNA in Tail*	8.8 ± 19.7	20.0 ± 23.8	19.7 ± 25.0	3.0	0.218(NS)

Kruskal–Wallis test, different letters = S, same letters = NS, P > 0.05 = NS, P < 0.01 = HS**, P < 0.05 = S *. Values are displayed as median with standard deviations ± SD (n = 9).

**Table 4:** Post hoc pairwise multiple comparison between groups' comet assay parameters

	Group (I)	Group (J)	Mean rank difference (I-J)	P value	Sig
*Tail length*	*Control Gp*	*SFZN Gp*	− 21.77	0.011 ^*^	S
		*ESOm Gp*	− 23.65	0.006 ^**^	HS
	*SFZN Gp*	*ESOm Gp*	− 1.88	0.827	NS
*Tail moment*	*Control Gp*	*SFZN Gp*	− 16.7	0.049 ^*^	S
		*ESOm Gp*	− 18.85	0.026 ^*^	S
	*SFZN Gp*	*ESOm Gp*	− 2.15	0.7997	NS

Dunn’s post hoc test, P > 0.05 = NS, P < 0.01 = HS**, P < 0.05 = S*

## Discussion

To the best of our knowledge, studying the histologic findings in the buccal mucosa due to the use of SFZN individually and in combination with ESOm has not been described previously. Thus, this study provides a novel histologic description of the effect of these drugs on buccal mucosa, providing a valuable nidus for future research.

In our study, male rats were chosen over female rats to avoid any possible modulatory effect of estrogen [22]. The buccal mucosa could be used to monitor early genotoxic events, and 90% of all cancers appear to be epithelial in origin [23].

The pyknotic nuclei and vacuolation detected in the SFZN Gp of our study are in line with the findings by Heikal et al. (2023) [24], which were detected in the renal tissue in response to SFZN administration and were attributed to increased reactive oxygen species (ROS), which could have been exaggerated in the SFZN group of our study due to the downregulated GSH levels, as proved by our investigations. Thepleomorphic nuclei detected in our study in SFZN Gp and ESOm Gp are similar to what was reported in epithelial cells of smokers [25].

The possible similarity between SFZN and smoking is that both have an impact on tissues through the occurrence of hypoxia, as smoking is related to high levels of serum carboxyhemoglobin [26], and SFZN is related to high levels of serum methemoglobin [27].

The nuclear changes were detected along the whole epithelial thickness of the SFZN Gp and were confined to the superficial layers in the ESOm Gp, which highlights the possible protective effect of ESOm on buccal tissue when administered in combination with SFZN.

The irregularities detected in the BM of SFZN Gp could be due to the altered architecture of the epithelium, visible in our histological results, as mentioned by Meinl and Brunner (1980) [28], and could be due to variable changes detected in our study, which require further investigations at the electron microscopic level to elucidate the nature of these irregularities.

Some of the histologic findings detected in SFZN Gp, including premature keratinization and increased keratin thickness, resemble the signs of architectural and cytological features of oral epithelial dysplasia described previously [29]. Our novel results offer valuable resources and insight for future research in the field, as these findings were restored in the ESOm Gp, which showed epithelial, LP, and BM architectures that were comparable to those of the control Gp.

Cell enlargement with marked rounding is commonly observed in most ferroptotic cells and iron deposition in the tissues is a hallmark of ferroptosis [4]. We found characteristic rounding of the basal cells and significant iron deposition, detected in the SFZN Gp and ESOm Gp according to the results of our study using Mallory Prussian blue special stain.

According to our observations, there was increased spacing between the collagen fibers in the SFZN Gp, which may reflect degradation of collagen fibers that were not observed in the control Gp. Collagen degradation and vacuolation occur post-mortem due to a lack of oxygen, leading to protein denaturation and collagen degradation [30]. Tissue hypoxia, as a subsequent result of hypoxemia resulting from methemoglobinemia [27], could be a reasonable explanation for the LP spacing detected in our results.

Scoring of the histological findings was not within the scope of our study as we relied basically on other parameters concerned with changes at the molecular level (GSH tissue level, iron deposition in the buccal mucosa, immunohistochemical reaction to Nrf2 levels and DNA degradation analysis). Further studies are required to correlate the results of our study with different histological scoring systems.

Nrf2 has been described as the superstar of ferroptosis [31]. Our results reflected downregulation of Nrf2 in the SFZN Gp, even significantly less than that detected in the ESOm Gp. This reflects that ESOm administration rescued the level of Nrf2 to some extent, reflecting that SFZN disables or has a negative regulation on the Nrf2 machinery, a hallmark of ferroptosis. This could explain the histological findings in the SFZN, reflecting impaired epithelialization represented by the excessive keratin thickness and increased number of stratum granulosum layers. Deficient Nrf2 mice have been found to show excessive keratinization [32].

However, these results are contradictory to the results published previously [33], which revealed that using SFZN with high glucose induced endothelial dysfunction reduced the damage in the vascular endothelium by activating various mechanisms, including the Nrf2 antioxidant pathway.

This finding may highlight that the structural difference between the epithelium and the endothelium might be the cause behind this contradictory finding, suggesting that a comparative study of the two tissues under the same circumstances is mandatory to elucidate the effect of SFZN.

The Nrf2 expression detected in ESOm Gp, which was statistically downregulated compared to the control group, could have been maintained through KEAP1 deactivation [32]. Further investigations are required to reveal the exact mechanism.

Depletion of GSH levels is a hallmark of ferroptosis [4] and carries disequilibrium of the cellular antioxidant defense mechanism [2]. The results of our study reflected a significant depletion of buccal tissue GSH in SFZN Gp. This aligns with what was reported previously [1], regarding renal GSH depletion due to the use of SFZN.

On the other hand, the depletion of GSH in the ESOm Gp compared to the control Gp was still significantly higher than that of the SFZN Gp. This reflects to some extent that ESOm, in combination with SFZN, rescued the GSH level, and further investigations are required.

A contradictory result that requires further investigations to reveal the exact mechanism is the significantly higher tissue iron deposition in the ESOm Gp in relation to SFZN Gp and to the control Gp.

The comet assay has been widely used in human biomonitoring to investigate the geno protective and genotoxic effects of various agents and was considered an easy method, especially of the buccal mucosa, as it has a proven sensitivity to detect low levels of DNA damage and is superior to other methods [20, 34].

The genetic makeup of eukaryotic organisms is encoded in double-stranded DNA. Single-stranded DNA, which is more vulnerable to damaging attacks by endogenous or exogenous chemicals, is important for the replication, recombination, and transcription processes; therefore, special single-stranded DNA-binding proteins participate in the protection of it [35]. Single-stranded DNA damage negatively affects the normal replication machinery [36]. We found single-stranded DNA damage in SFZN Gp and ESOm Gp, which reflects the hazardous effect of these drugs at the nuclear level.

Single-stranded DNA damage can activate certain damage response mechanisms, triggering cell cycle checkpoints and preventing potentially damaging DNA propagation [36]. Therefore, further research and follow-up studies are needed to reveal the association between single-stranded DNA damage detected in our study and possible repair mechanisms to deeply investigate the exact extent and effect of SFZN and ESOm on DNA damage and to better interpret the results of our study and its clinical relevance.

Moreover, a future study using neutral comet assay conditions and multiple software packages rather than a single software would be recommended to yield a more accurate interpretation of the results.

The aforementioned data highlight the importance of further future investigation of the NRF2\KEAP1 signaling pathway and its exact relation to using SFZN and ESOm at different concentrations and durations.

## Conclusion

In our study, we conclude that hallmarks of ferroptosis occur in the buccal mucosa in response to the use of SFZN individually and in combination with ESOm, with tissue iron deposition, depletion of GSH to variable degrees, marked effect on the Nrf2 machinery, and single-stranded DNA damage carrying potential hazardous effects at the nuclear level. In addition, using SFZN individually caused defective epithelialization and increased keratin thickness at the histological level. Thus, this provides a possible explanation for expected oral side effects upon using SFZN and giving deep insight into potential management.

## Data Availability

The corresponding author can be contacted to provide the data of this study on reasonable request.

## Abbreviations

BVs: Blood vessels
ControlGp: Control group
ESOm Gp: Esomeprazole group
ESOm: Esomeprazole
GSH: Glutathione
LP: Lamina propria
NRF2: Nuclear factor erythroid two-related factor 2
OL: Outline
ROS: Reactive oxygen species
SFZN Gp: Sulfasalazine group
SFZN: Sulfasalazine
SSD: Statistically significant difference
